# MiR-146a Restoration Suppresses Triple-Negative Breast Cancer Cell Migration: A Bioinformatic and *In Vitro* Study

**DOI:** 10.34172/apb.2022.083

**Published:** 2021-10-06

**Authors:** Leila Tebbi, Behzad Mansoori, Sahar Safaei, Shahriar Hashemzadeh, Masoud Shirmohamadi, Khalil Hajiasgharzadeh, Mahdi Abdoli Shadbad, Behzad Baradaran

**Affiliations:** ^1^Immunology Research Center, Tabriz University of Medical Sciences, Tabriz, Iran.; ^2^Department of Cancer and Inflammation Research, Institute for Molecular Medicine, University of Southern Denmark, Odense, Denmark.; ^3^Student Research Committee, Tabriz University of Medical Sciences, Tabriz, Iran.; ^4^Department of Thoracic Surgery, Tabriz University of Medical Sciences, Tabriz, Iran.; ^5^Liver and Gastrointestinal Diseases Research Center, Tabriz University of Medical Sciences, Tabriz, Iran.; ^6^Connective Tissue Diseases Research Center, Tabriz University of Medical Sciences, Tabriz, Iran.; ^7^Neurosciences Research Center, Tabriz University of Medical Sciences, Tabriz, Iran.

**Keywords:** MicroRNAs, Breast neoplasms, Transfection, Cell proliferation, Wound healing

## Abstract

***Purpose:*** Breast cancer is one of the most commonly diagnosed types of cancer worldwide. This cancer is treated with various methods like mastectomy, chemotherapy, hormone therapy, and radiotherapy. Among them, targeted therapy, like microRNA (miRNA) replacement therapy, is considered a new approach to treating breast cancer.

***Methods:*** Data analysis from TCGA datasets were used to investigate the expression of hsa-miR-146a-5p in breast cancer. MTT assay was used to evaluate the viability of MDA-MB-231 cells after hsa-miR-146a-5p ectopic expression. A wound-healing assay was used to observe migration in the MDA-MB-231 cell line and the effect of the hsa-miR-146a-5p ectopic expression on migration. Finally, quantitative reverse transcription polymerase chain reaction (qRT-PCR) was used as a method to determine the effect of the hsa-miR-146a-5p ectopic expression on the expression of CXCR4, β-catenin, MMP2, MMP9, and Vimentin genes known to be involved in invasion and migration of MDA-MB-231 cells.

***Results:*** Our results indicated that hsa-miR-146a-5p is not involved in apoptosis in the MDAMB-231 cells, while it is highly effective in migration inhibition. MMP9, MMP2, CXCR4, and Vimentin expressions were suppressed by hsa-miR-146a-5p induction; however, it induced the expression of β-catenin.

***Conclusion:*** Some non-coding RNAs, such as hsa-miR-146a-5p, are effective in breast cancer targeted therapy. As cancer is a complicated disorder, therefore the combination of therapies might lead to novel therapeutic strategies.

## Introduction

Among cancer patients, breast cancer is one of the fatal cancers.^1,2^ Based on the data from public health reports, over one million women are diagnosed with breast cancer throughout the world every year, which leads to death in 50% of the cases.^3^ Also, according to reports worldwide, it seems that the incidence of breast cancer has been rising yearly.^3^ In Iran, breast cancer is the first diagnosed cancer among women. Over the last few decades, several studies have been conducted on breast cancer in Iran, and the results have indicated an increased rate of breast cancer.^4^ There are different therapeutic approaches to treat breast cancer such as mastectomy,^5,6^ chemotherapy,^7,8^ hormone therapy,^9,10^ and radiotherapy.^11,12^ In recent decades, different treatments have been used to increase the efficacy of these methods in patients, such as chemotherapy, which has been used as an adjuvant therapy to mastectomy.^13^ Another example is the combination of chemotherapy along with a monoclonal antibody.^7^

Among patients with breast cancer, it seems patients treated with chemotherapy experience more side effects. The chemotherapy drugs, along with the cancerous cells, also attack the healthy cells. This has become the main reason for researchers over the last decades to try and find agents like miRNA that affect cancerous cells via targeting the oncogenic mRNA at the posttranscriptional level.^14,15^ The most important step in targeted therapy is to determine the right target, which is only possible by a vast knowledge of the molecules and proteins involved in cancer.^16^ Choosing the right target will keep the healthy cells safe and increase life expectancy in patients.^17^ A type of small non-coding RNAs that includes about 22 nucleotides, called miRNAs, are considered important regulators with crucial roles in cancer, such as regulating gene expression, metastasis, tumor suppression, and oncogenesis.^18-20^ Due to the regulatory role of miRNAs, any altered expression of them may lead to cancer development.^21-23^ MiR-146a is one of the miRNAs that has been reported as a molecule that could be involved in different cancers like prostate, gastric, and breast cancer due to dysregulations.^24,25^ Therefore, restoring these molecules in patients is considered an important therapeutic strategy.^21,22^ Studies have shown that miR-146a targets different genes, including MMP9, MMP2, vimentin, β-catenin, and CXCR4,^26-29^, which are known to be involved in metastasis and invasion in breast cancer.^30-33^ Overall, in this research, we aimed to investigate the role of miR-146a in breast cancer by using bioinformatic analysis and *in vitro* assays, including cytotoxicity assay, wound healing assay, and apoptosis assays. Also, we analyze the expression levels of the genes relating to tumor migration, such as MMP2, MMP9, CXCR4, β-catenin, and vimentin in MDA-MB-231 cell lines after miR-146a ectopic expression to ultimately determine the effect of miR-146a on the migration of cancer cells.

## Materials and Methods

###  Bioinformatic analysis

The breast cancer clinical data were extracted from The Cancer Genome Atlas-Breast Cancer (TCGA-BRCA) project. We accessed the TCGA-BRCA data using the UCSC Cancer Browser (https://xenabrowser.net/). We compared the hsa-miR-146a-5p expression in breast cancer and normal breast tissues.

###  Breast cancer cell culture

The MDA-MB-231 cell line was purchased from Pastor Institute in Iran (Iran, Tehran) and cultured in RPMI-1640 medium (Gibco, Lot No. 1703986X), supplemented with 10% Fetal Bovine Serum (FBS) (Gibco, Lot NO. 42F8160K, USA). The medium was later added 1% antibiotic (penicillin 100 IU/ml, streptomycin 100 µg/ml) and sterilized with 0.22-micron filters. After de-freezing, cells were transferred to a T25 cell culture flask. The flask was kept in the incubator at 37°C, with 5% CO_2_, and 60% humidity. The media was changed every 2 days until the cells entered the logarithm phase. Then they were ready to be used for the tests according to our previous study.^34^

###  MiR-146a ectopic expression

Cells were seeded into a 6 well plate; 2×10^5^ cells in each well, and incubated for 24 hours. After 24 h and as the first step, cells were transfected by miR-146a in a 6 well plate to set the optimum concentration.^35^ We used 4 µL transfection reagent (TR) (JetPrime, PolyPlus, France) for each well with different doses of miR-146a and optimum media, which was 1 pmol miRNA and 99 µL media for first well, 5 pmol miRNA plus 95 µL media for the second well, 10 pmol miRNA and 90 µL media in triplicate. As the second step, the cells were seeded in a 6 well plate and transfected by 10 pmol miR-146a, which was determined as the optimal dose before this test. The cells were incubated for 24 and 48 to determine the optimum time.

###  Quantitative real-time polymerase chain reaction ( qRT -PCR)

####  RNA extraction (isolation)

 The total amount of RNA was isolated from each well using RiboEx reagent (Gene All ، Lot NO. REX15J12014, Korea) and transferred into a microtube. Based on the protocol, 120 µL chloroform (Merck, Germany) was added to each microtube and was kept at -20°C for 10 minutes. Then the mixture was centrifuged for 20 minutes at 12000 rpm and 4°C. The surface layer, which is the aqueous phase and contains RNA, was collected and mixed with 250 µL cold isopropanol. Then, it was left at -20°C for 45 minutes. The mixture was centrifuged again for another 20 minutes at 4°C and 13000 rpm. The surface layer was thrown out, and the residue was added to 500 µL cold 75% ethanol and was centrifuged for 10 minutes at 7800 rpm and 4°C. The last step was repeated one more time. As the final step, the surface layer was thrown out, and the microtubes were dried out in the dry bath at 56°C for 10 minutes after it was added 30 µL Nuclease free water solution (Lot No. 00181198, EXIQON, Denmark). Extracted RNA was stored at -80°C upon analysis.

####  cDNA synthesis

 First, the OD of RNA samples were calculated using NanoDrop 2000c (Thermo, USA). After that, cDNA was synthesized by the EXIQON kit. Based on the protocol, 5 µg/µL RNA was needed for cDNA synthesis. Afterward, 2µL 5x reaction buffer (EXIQON Lot NO.176643, Denmark), 1 µL nucleic mix, and 1 µL reverse transcriptase were added to a microtube. The temperature protocol includes 42°C for 60 minutes, 95°C for 5 minutes, and 4°C for storage. cDNA for mRNA performed by biofact kit (Seoul, South Korea) according to manufacturer protocols. Briefly, 5 µg/µL total RNA, 1 µL Oligo (dT), 1 µL random hexamer primers, and 10-enzyme mixture were added to the 0.1 µL microtube. The temperature protocol includes 25°C for 5 minutes, 42°C for 60 minutes, 85°C for 5 minutes, and 4°C for storage. The microtubes were later transferred to a thermal cycler (BioRad model T100 Thermal Cycle, SN 621BR141187, USA).

####  qRT-PCR

After that, samples were analyzed by qRT-PCR. First, 4 µL cDNA diluted 1 to 80 were transferred to PCR microtubes. Second, a 5 µL master mix was also added to microtubes. The master mix is a premixed concentrated solution of SYBER green (EXIQON Lot No. 203421, Denmark), dNTP, 1 µL primer, and U6. U6 is used as the internal control. 1 µL hsa-miR-146a (EXIQON ID: 204483, Denmark) was also added to the mixture. Finally, we put the microtubes in Light Cycle 96 (Roche REF: 05815916001 J SN: 11769 Germany). All three steps were programmed according to the protocol, which was 95ºC for 10 seconds in the first step, 60ºC for 60 seconds in the second step, and melting in the final step. We also used qRT-PCR to determine whether the expression levels of MMP9, MMP2, vimentin, B-catenin, and CXCR4 (Sinaclon, Iran) were affected by miR-146a (Microcynth, Switzerland) ectopic expression. We used 18s rRNA (Sinaclon, Iran) as an internal control. The sequences of the primers are represented in Table 1.

**Table 1:** miR‐146a and primer sequences in real-time PCR

**Target gene**	**Strand**	**Sequence**
Hsa‐miR‐146a		5'-UGAGAACUGAAUUCCAUGGGUU-3'
U6	Forward	5'-GGCAGCACATATACTAAAATTGG-3'
	Reverse	5'-AAAATATGGAACGCTTCACGA-3'
MMP2	Forward	5'-GCCCTCCTGGGAATGAAGCAC-3'
	Reverse	5'-GCATTGCCTCTGGACAACACA-3'
MMP9	Forward	5'-ATTCATCTTCCAAGGCCAATCC-3'
	Reverse	5'-CTTGTCGCTGTCAAAGTTCG-3'
CXCR4	Forward	5'-TCTTCCTGCCCACCATCTACTC-3'
	Reverse	5'-TGCAGCCTGTACTTGTCCGTC-3'
Vimentin	Forward	5'-CAGGCAAAGCAGGAGTCCA-3'
	Reverse	5'-AAGTTCTCTTCCATTTCACGCA-3'
β-catenin	Forward	5'-CACAAGCAGAGTGCTGAAGGTG-3'
	Reverse	5'-GATTCCTGAGAGTCCAAAGACAG-3'
18s	Forward	5'-GCTTAATTTGACTCAACACGGGA-3'
	Reverse	5'-AGCTATCAATCTGTCAATCCTGTC-3'

###  Cytotoxicity assay

MTT assay was used to evaluate the viability of cells. In this method, we used Tetrazolium salt. We seeded 15×10^3^ MDA-MB-231 cells in a 96 well plate and incubated them for 24 hours. After the passing time, cells were transfected in a triplicate way for more accuracy with three dosages of miRNA-146a (1 pmol, 5 pmol, 10 pmol) for 24 hours. Then, 50 µL MTT solution (2 mg/mL Bio Basic, Lot No. DU21373R2, Canada) was added to each well, and plates were incubated for 4 hours. After 4 hours, the surface layer was thrown out, and the cells were washed with 100 µL phosphate-buffered saline (PBS) (Gibco, Lot No. 2259817, USA). Next, 200 µL dimethyl sulfoxide (DMSO) was added to each well, and plates were incubated at 37°C for 30 minutes. The values of the optical density of the cells were evaluated at 570 nm with an ELISA Reader (Sunrise RC, Tecan, Switzerland).

###  Scratch test (wound healing assay)

Cells were seeded in 2 wells of a 12-well plate with approximately 15×10^4^ cells in each well. Cells were then incubated for 72 hours to reach the right confluency. One well was transfected by 10 pmol (optimum dosage) of miR-146a. Before transfection, we created a wound gap in the bottom of the plate using the tip of a yellow micropipette. Then, we started taking photos at once. The first photo was taken immediately after transfection (time point 0), and the others were taken after 24 and 48 hours to determine the migration of the MDA-MB-231 cells following hsa-miR-146a-5p ectopic expression.

###  DAPI staining

 First, 5000 cells were seeded in a 96-well plate. Later, the cells were transfected with the optimum dosage of miR-146a (10 pmol resulted from qRT-PCR). 24 hours later, the media was thrown out, and cells were washed with PBS. Next, 200µL paraformaldehyde 4 % was added to each well, and plates were incubated for 60 minutes to fix the cells. After that, the cells were washed with PBS and then added 200 µL triton 0.1% and were left at room temperature for 10 minutes to reduce the surface tension. Finally, the cells were washed with 200 µL PBS and stained with DAPI (4′, 6-diamidino-2-phenylindole). This fluorescent color that binds to DNA in adenine- thymine enriched regions, enters the cells through the cell membrane; thus, it is an efficient way to observe the viable cells. 10 minutes later, the solution was aspirated and 200 µL PBS was added, and the plate was taken to the imaging system (Biotek).

###  Flow cytometry

 After miR-146a ectopic expression, annexin V/propidium iodide (PI) assay was used to study apoptosis in MDA-MB-231 cells. The microtubes were divided into two groups: control groups and transfected by the miR-146a group. In brief, the detached cells were centrifuged at 1300 rpm for 5 minutes. Then, the cells were stained with an Annexin V‐FITC/PI staining assay kit according to the manufacturer’s protocol (Roche). After that, microtubes were kept at RT for 15 minutes in the dark. Both groups were evaluated by the flow cytometer instrument (MACS Quant 10; Miltenyi Biotec, GmbH, Germany). Later, FlowJo software (Tree Star, San Carlos, CA) was used to evaluate the apoptosis rate.

###  Statistical analysis

All data are shown as the mean ± standard error of the mean (SEM). GraphPad Prism 6 software (San Diego, CA, USA) was applied for statistical analysis. One-way analyses of variance were done to demonstrate statistical differences among more than two groups. The *P* values smaller than 0.05 were considered statistically significant.

## Results and Discussion

To the extent of our knowledge from this research and other related researchs, miR-146a has been known to be a metastasis suppressor^36-38^ in different cancers like prostate and gastric cancer.^39,40^ However, the effects of miR-146a-5p on cancer-related properties in the MDA-MB-231 cells are unclear. Here, we conducted a bioinformatic analysis and a set of different *in vitro* experiments to determine its role in MDA-MB-231 development.

###  Bioinformatics analysis of miR-146a expression in breast cancer samples

**Figure 1:**
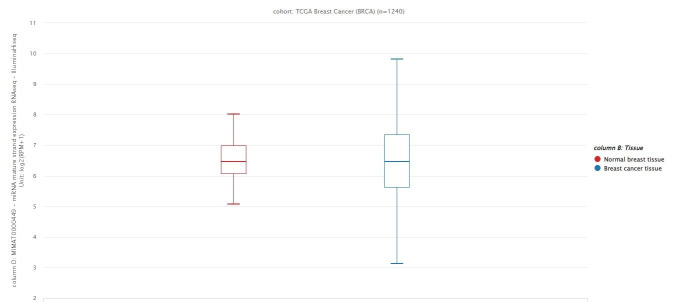
The data extracted from the BRCA project from the TCGA dataset.

According to the data extracted from the TCGA dataset BRCA project, there has been no significant difference in hsa-miR-146a-5p expression between breast cancer tissues and normal breast samples (Figure 1).

###  Optimal miR-146a ectopic expression was the dose-dependent manner in 48 hours

**Figure 2:**
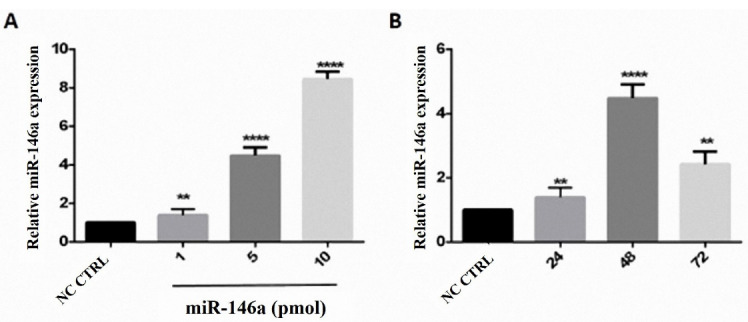
hsa-miR-146a-5p ectopic expression in a dose-dependent manner. A) The highest level of miR-146a was displayed at 10 pmol concentration. B) The optimal time for miRNA induction was 48 h after transfection. **P value <0.01, *** *P* value <0.001, and **** *P* value <0.0001

Quantitative real-time PCR was used after transfecting cells with 1 pmol, 5 pmol, and 10 pmol of miR-146a to determine the optimum dosage based on the expression of miR-146a. The optimum dosage of miR-146a was determined10 pmol. The optimum time was 48 hours (Figure 2).

###  MiR-146a was not able to decrease cell viability

**Figure 3:**
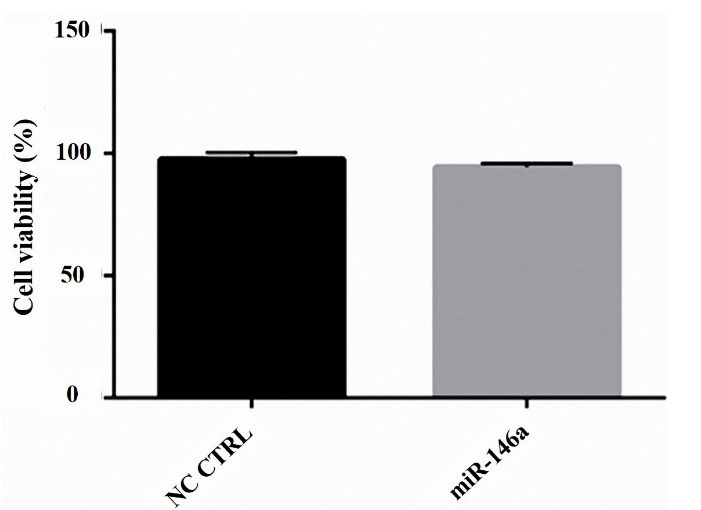
hsa-miR-146a-5p ectopic expression did not have cytotoxicity on MDA-MB-231 cells

The MTT assay was done to show cell viability. We realized that miR-146a ectopic expression has no effect on cell viability in the MDA-MB-231 cells (Figure 3).

###  MiR-146a is not involved in cell apoptosis

**Figure 4:**
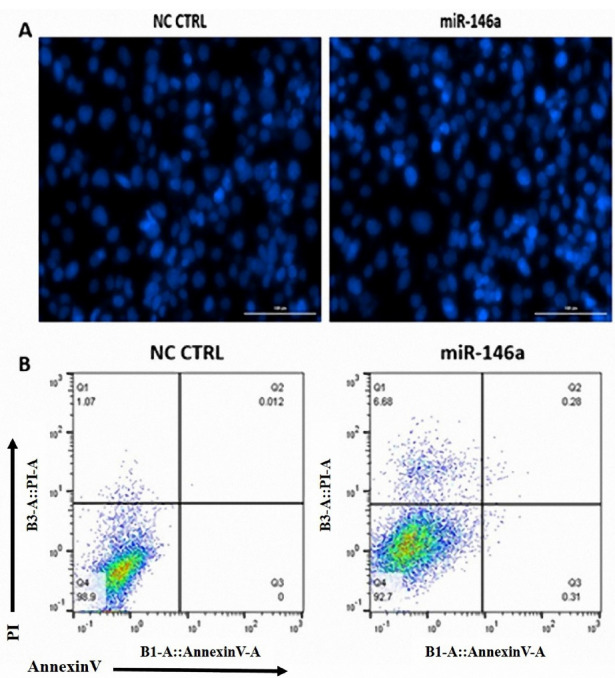
hsa-miR-146a-5p ectopic expression does not induce apoptosis in MDA-MB-231 cells. (A) DAPI staining showed no significant change in MDA-MB-231 nucleus fragmentation following hsa-miR-146a-5p ectopic expression. (B) AnnexinV/PI apoptosis assay did not show any significant change in early and late apoptosis following hsa-miR-146a-5p ectopic expression

DAPI staining and annexin V/PI assays were performed to show the effect of miR-146a on apoptosis in MDA-MB-231 cells. According to our results, miR-146a ectopic expression does not induce apoptosis; nucleus fragmentation was not visible in the images (Figure 4A), and there was no change between in early and late apoptosis flow cytometric quadrants (Figure 4B).

###  MiR-146a suppressed breast cancer cell migration

**Figure 5:**
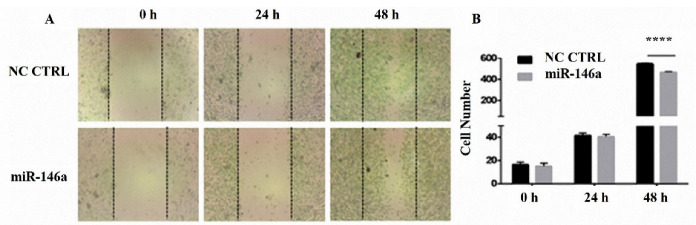
hsa-miR-146a-5p ectopic expression inhibited MDA-MB-231 cells migration. (A) The images of scratched areas of control and transfected with hsa-miR-146a-5p at 0, 24, and 48 h after the scratch mark. (B) Numbers of the migrated cells in the scratched area. **** *P* value <0.0001

The wound-healing assay was performed to investigate migration in the MDA-MB-231 cells. The results were gathered by images. Based on the results, MDA-MB-231 cell migration was inhibited after 48 hours of miR-146a transfection (Figure 5).

###  MiR-146a decreased MMP9, MMP2, and CXCR4 expression

**Figure 6:**
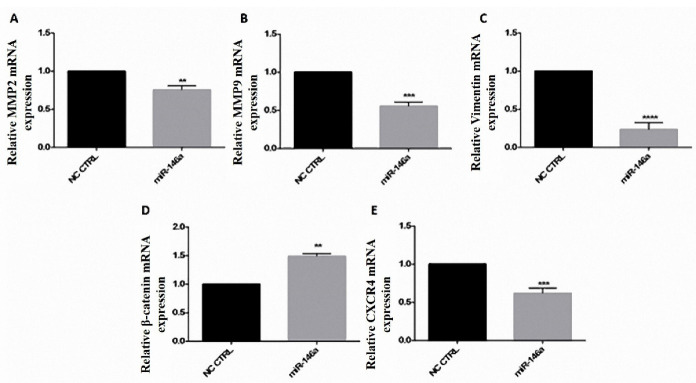
hsa-miR-146a-5p ectopic expression reduced migration-related genes in breast cancer cells. A-E) hsa-miR-146a-5p ectopic expression decreased MMP2, MMP9, Vimentin, and CXCR4 mRNA expression; hsa-miR-146a-5p ectopic expression increased β-catenin mRNA after transfection. **P value <0.01, *** *P* value <0.001, and **** *P* value <0.0001

MiR-146a downregulated the expression of MMP9, MMP2, CXCR4, vimentin, and upregulated β-catenin expression. MMP9 was reduced to 0.5 fold, CXCR4 to 0.6 fold, MMP2 to 0.8 fold, and vimentin to 0.2 fold; However, β-catenin was increased to 1.5 fold. Overall, a reduction in cell migration was observed (Figure 6).

Similar to our results, in a study by Hurst et al., miR-146a was upregulated by breast cancer metastasis suppressor 1, as an inhibitor of metastasis of cancerous cells. This research has indicated that miR-146a, an inhibitor of invasion and migration and metastasis, reduced breast cancer migration into the lung tissue.^41,42^ Tao et al. reported the induction of apoptosis by miR-146a upregulation under the effect of quercetin in the MDA-MB-231 cell line in 2015^43^; however, the results from this research showed that hsa-miR-146a-5p is not involved in the process of apoptosis in MDA-MB-231 cell lines. It was also shown that transfecting MDA-MB-231 cells that were treated with quercetin by miR-146a inhibit cell growth.^43^ In contrast, we concluded in this study that cell viability is not affected by miR-146a ectopic expression. In the present study, we investigated the expression level of MMP9, MMP2, CXCR4, and vimentin, genes involved in metastasis in breast cancer. We indicated a significant relationship between the expression of these genes and the level of expression of miR-146a. Based on our data, CXCR4 expression is downregulated by the overexpression of miR-146a.^44^ The previous studies on CXCR4 have pinpointed that CXCR4 is a metastasis inducer; thus, its down-regulation leads to inhibition of metastasis.^45,46^ MMP2 and MMP 9 are considered important factors in the induction of metastasis.^47,48^ In this research, our findings were consistent with other studies on metastasis in breast cancer, which detected an increase in the expression of MMP2 and MMP 9 in breast cancer.^33,49^ As stated, transfection of MDA-MB-231 cells by miR-146a reduced the expression of MMP2 and MMP9.^50^

**Figure 7:**
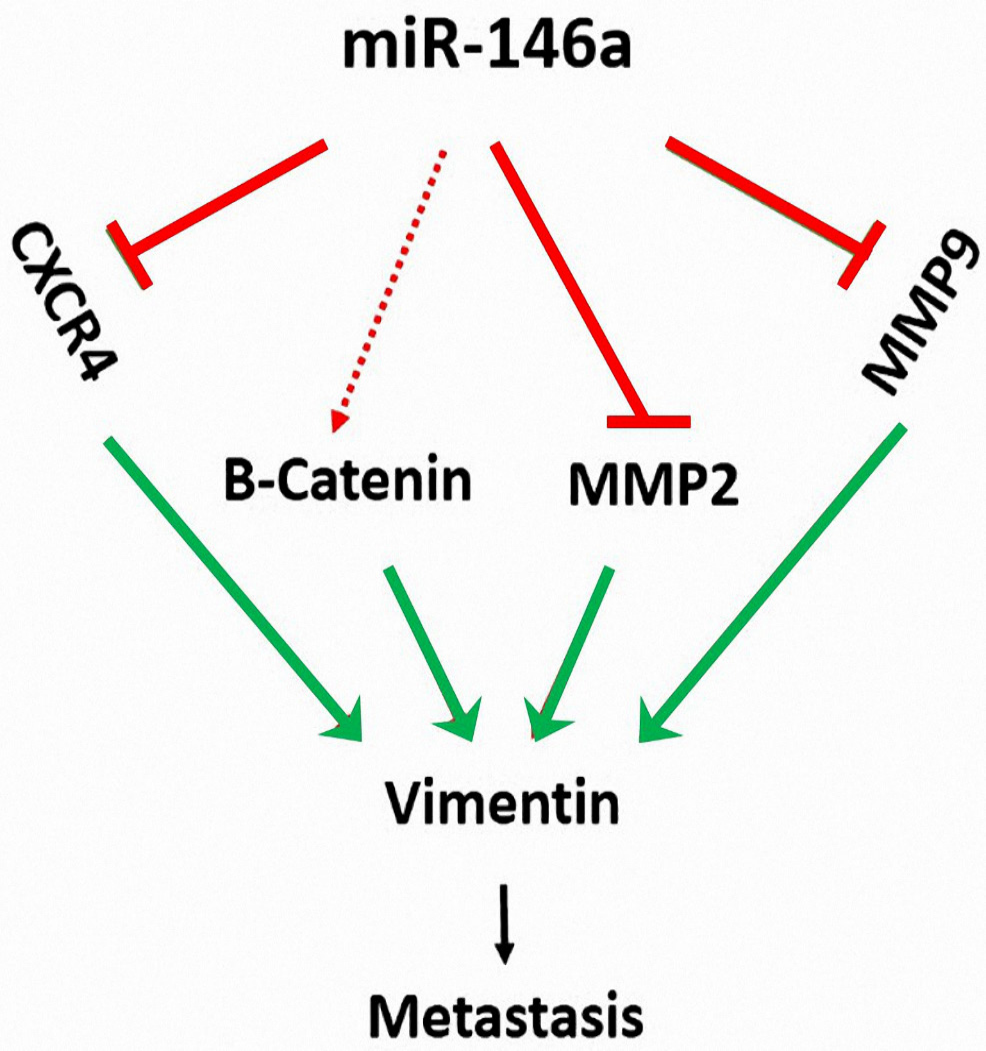
hsa-miR-146a-5p ectopic expression reduces the expression of MMP2, MMP9, and CXCR4 and inhibits the migration of MDA-MB-231 cells

It has been reported that β-catenin can regulate metastasis in TNBC.^51^ Vimentin overexpression is an important factor in increased metastasis in different cancers^52,53^ including breast cancer.^54^ Among all the genes, vimentin was reduced the most, which led us to conclude that its expression is highly affected by miR-146a induction. Overall, vimentin is highly involved in malignant cell migration.^30^ (Figure 7).

## Conclusion

In summary, this study indicated that hsa-miR-146a-5p is a metastasis inhibitor in the MDA-MB-231 cell line. Consequently, it could be a therapeutic strategy to inhibit metastasis in breast cancer by targeting the genes above. Sun et al. have studied miR-146a and miR-146b in papillary thyroid carcinoma.^55^ Li et al. have studied miR-146a in pancreatic cancer development.^39^ Our findings indicate miR-146a as a candidate for future microRNA replacement therapy to inhibit the metastasis in breast cancer.

## Acknowledgments

 We appreciate the researchers of the Immunology Research Center, Tabriz University of Medical Sciences, Tabriz, Iran.

## Ethical Issues

 All experiments and procedures were conducted in compliance with the ethical principles of Tabriz University of Medical Science, Tabriz, Iran and approved by the regional ethical committee for medical research (Ethical code: IR.TBZMED.REC. 1398.321).

## Conflict of Interest

 The authors have no conflicts of interest to declare.
